# Supplementary material for: Development of a multivariate predictive model for dapsone adverse drug events in people with leprosy under standard WHO multidrug therapy
Source: PLoS Negl Trop Dis. 2024 Jan 25;18(1):e0011901. doi: 10.1371/journal.pntd.0011901 (PMC10846698; doi:10.1371/journal.pntd.0011901)
Supplement: S1 Table — (DOCX) [file pntd.0011901.s001.docx]

**Supporting information**

**S1 Table. Other comorbidities evaluated of 329 leprosy patients treated at Souza Araujo outpatient clinic (ASA), Rio de Janeiro, Brazil between 2000-2021.**

|  |  | Cases | Controls | P-value^a^ | OR (95% CI) |
| --- | --- | --- | --- | --- | --- |
| Smoking  habits | Yes  No | 23 (19,17%)  93 (77,5%) | 40 (19,14%)  160 (76,56%) | 0.97 | 1  1 (0.57-1.81) |
| Arterial hypertension | Yes  No | 24 (20%)  92 (76,67%) | 50 (23,92%)  151 (72,25%) | 0.396 | 1  1.27 |
| Diabetes mellitus | Yes  No | 12 (10%)  105 (87,5%) | 18 (8,61%)  182 (87,08%) | 0.712 | 1  0.86 (0.4-1.92) |
| Cardiovascular disease | Yes  No | 6 (5%)  110 (91,67%) | 13 (6,22%)  188 (89,95%) | 0.64 | 1  1.25 (0.47-3.71) |
| Respiratory disease | Yes  No | 8 (6,67%)  109 (90,83%) | 11 (5,26%)  189 (90,43%) | 0.628 | 1  0.79 (0.31-2.13) |
| Kidney disease | Yes  No | 5 (4,17%)  113 (94,17%) | 11 (5,26%)  190 (90,91%) | 0.626 | 1  0.78 (0.23- 2.23) |
| Background Tuberculosis | Yes  No | 4 (3,33%)  113 (94,17%) | 11 (5,26%)  189 (90,43%) | 0.4 | 1  1.6 (0.53-6.09) |
| HIV | Yes  No | 2 (1,67%)  113 (94,17%) | 7 (3,35%)  179 (85,65%) | 0.316 | 1  2.09 (0.48-15.73) |

^a^ Pearson’s Chi-squared test

OR, odds ratio; CI, confidence interval;
