# Supplementary material for: Development of a multivariate predictive model for dapsone adverse drug events in people with leprosy under standard WHO multidrug therapy
Source: PLoS Negl Trop Dis. 2024 Jan 25;18(1):e0011901. doi: 10.1371/journal.pntd.0011901 (PMC10846698; doi:10.1371/journal.pntd.0011901)
Supplement: Attachment — Submitted filename: Carta ao editor - revisores artigo.docx [file pntd.0011901.s002.docx]

Rio de Janeiro, November 12^th^ 2023:

To: Dr. Peter J.Hotez MD, PhD

Editor of PLOS Neglected Tropical Diseases

Dear Dr Peter J.Hotez MD, PhD

We would like to thank for the critical evaluation of our manuscript PNTD-D-23-01034 entitled “Development of a multivariate predictive model for dapsone adverse drug events in people affected by leprosy under standard WHO multidrug therapy”. As requested, we are now returning the revised versions of the manuscript, together with a point-by-point response letter in which we tried to provide answers for all questions raised by the referees. All the changes made in the text of the revised manuscript (“highlights changes” version) were highlighted in red.

Finally, we hope that after these changes the revised manuscript would be suitable for publication in the PLOS Neglected Tropical Diseases.

Sincerely yours,

Ana Carolina Galvão dos Santos de Araujo.

Corresponding author

Dear Referees:

Thank you for reviewing our paper so carefully. Your comments and suggestions were pertinent and greatly contributed to improving the text, which has been revised to include your suggestions and answer your questions, as follows:

**Answers to REVIEWER 1 comments:**

***General comments***

***The manuscript has interesting scope, and it contains valuable data. Some modifications are needed:***

***1. Please recheck all abbreviation and their full name for first time in the manuscript. For example, line 105 GWAS????***

***2. Please change all "dapsone" to "DDS" in the manuscript, because you used this abbreviation for first time.***

***3. I suggest you provide an abbreviation list after abstract section***

***4. Please add the limitations of the study at the end of manuscript.***

***Answer:*** Based in the reviewer suggestions we checked and changed abbreviations mistakes, we included an abbreviation list at the end of the abstract section, as suggested, and we highlighted our limitations. Please verify the new version of the manuscript.

**##################################################################**

**Answers to REVIEWER 2 comments:**

***General comments***

***Need for acknowledgement of the weaknesses of the study.***

**Answer:** Based in the reviewer suggestions we highlighted our limitations. Please verify lines 388-397 in the new version of the manuscript.

***Results***

***The results are clearly described. However, it is not clear whether the results are at all surprising or indeed very useful. Table 4 suggests that the only significant predicters are female sex, MB disease and having completed primary education (the last factor may be an artifact, with educated people being more ready to complain about a possible ADE.***

***Answer:*** Thank you for your valuable feedback. We believe that our model is an example of tool that can assist clinicians in identifying which patients are more likely to develop adverse drug events to dapsone. However, we acknowledge that further validation, particularly through external means and with the inclusion of laboratorial data, is necessary to ensure a more effective clinical application. However, we cannot exclude the fact that in some leprosy endemic areas laboratorial settings are limited and the identification of sociodemographic and clinical characteristics, such as gender, age, race, education status, clinical form of disease manifestation, and comorbidities that can predict ADEs can contribute for the development of strategies to minimize MDT complications.

***Conclusion***

***While the authors describe a nomogram to predict ADEs in patients taking MDT, they do not develop it into a simple rule-of-thumb that can be used at the bedside. To be useful, it must be simple and quick to use.  As mentioned above, this nomogram is of questionable usefulness, as it will simply pick out women with MB leprosy.***

***A more useful approach to ADEs in relation to MDT, which mainly involve dapsone, would be to suggest proactive steps that health staff may take to prevent some ADEs and identify others at an early and managable stage.***

***A recent publication on this topic should be referenced: Robin Hilder, Diana Lockwood; The adverse drug effects of dapsone therapy in leprosy: a systematic review; Leprosy Review; 2020; 91; 3; 232-243; DOI: 10.47276/lr.91.3.232
The authors seem happy to emphasize the strength of their study, but they omit to mention any limitations, which include the retrospective design, the lack of many laboratory results and the low practical value of the nomogram they developed.***

***Answer:*** Thank you for the insightful recommendation. Nomograms allow the integration and synthesis of the relative importance of clinical variables. Although nomograms cannot be a substitute for clinical judgement, they can optimize the individualized risk assessments for patients with ADEs. Although the absence of laboratorial data, which could improve the accuracy of the proposed nomogram, we believe the data provided here could help clinicians in identifying the sociodemographic profiles associated to ADEs development. As suggested, we incorporated in the discussion the findings from the publication by Hilder and Lockwood (line 393). Additionally, we have considered our study limitations as highlighted it. Please verify in the new version of the manuscript.

**##################################################################**

**Answers to REVIEWER 3 comments:**

***General comments***

***I suggest that the authors provide more robust information about the main topic of the study: predictive modelling. In the Introduction section, it is necessary to explore this further, in addition to the issues related to the drug and adverse events. The following can be considered as guiding questions: 1) How can the proposed predictive modelling contribute to clinical and epidemiological practice? 2) Why is it important to propose this predictive modelling? Furthermore, in the last paragraph of the Introduction section, it is important to define the objective of the study: "The objective of this study is to propose predictive modelling..." instead of mentioning that it was done. In the Methods section, it is important to clearly define the type of study: cohort or case-control. The Results section is well reported. In the Discussion section, authors need to indicate how the findings will contribute to clinical and epidemiological practice, as well as give more prominence to the proposed predictive modelling.***

**Answer:** Thanks for the comments. We made substantial changes in the new version of the manuscript. We clarify the objective of the study and have explicitly defined as a case-control study in the Methods section. Furthermore, we discussed how the model will contribute to both clinical and epidemiological practice (lines 395-405).

***Methods***

***The objective of the study was not clearly defined in the Introduction section. Also, the Introduction section does not contain enough context and justification about the proposed predictive modelling. But, in the Methods section, it was well articulated with a clear testable hypothesis stated. About the study design, I suggest that the authors define clearly, in the Methods section, if is a cohort or a case-control study. Beside this, the report address the stated objective. The population was clearly described and appropriate for the hypothesis being tested. As the authors included an entire cohort, the sample size is sufficient to ensure adequate power to address the hypothesis being tested. Correct statistical analysis were used to support the conclusions and there is sufficient report about ethical or regulatory concerns.***

**Answer:** Thanks for the comments. We clarify the objective of the study and have explicitly defined as a case-control study in the Methods section.

***Results***

***The analysis presented match the analysis plan. The results were clearly and completely presented. About the figures, I recommend that footnotes are included so that the tables and images are self-explanatory.***

**Answer:** As suggested, we added a detailed explanation on how the nomogram works in the figure legend (line 283).

***Conclusion***

***The conclusions are supported by the data presented. The limitations of analysis are clearly described. The authors discuss how these data can be helpful to advance our understanding of the topic under study, but I missed better details on how the proposed predictive model can be useful in clinical or epidemiological practice. I suggest that the authors provide more details on the main topic of the study: the proposed multivariate predictive model.***

**Answer:** We added in the Discussion section how the model will contribute to both clinical and epidemiological practice (lines 395-405), but as observed in the conclusion section, we highlighted the fact that this is a retrospective study that will need a prospective arm to validate and discuss the introduction of this tool in the clinical practice.

**##################################################################**

**Answers to REVIEWER 4 comments:**

***General comments***

***This is a most welcome study, and its results are robust and quite usefeull. Leprosy is a disease with scarce therapeutic choices and the prediction of adverse effects with MDT (the universal acceptable treatment for leprosy) is of utmost importance. If the prediction is made by means of easely identifiable criteria such as social and clinical characteristics, as is the case proposed in this study, the article assumes real importance to improve clinical care of cases. The results can help doctors at any level (including primary health care) to be aware of the possibility of adverse effects and they can mark theses as a special group needing special attention and, for instance, reducing the lag time between clinical revision. In this sense I do recommend to the editor to consider publishing this article.***

**Answer:** Thanks for the comments. We appreciate.

***No need for modifications, apart from Figure 2 (ROC curve) that seems not quite necessary for the general reader and could be removed. Of course, this is a decision of the Editor.***

**Answer:** Thank for the comment. ROC curve is a graphic representation of how accurate the model is, and we recommend to maintain it.
